# Dark fermentative hydrogen production and transcriptional analysis of genes involved in the unicellular halotolerant cyanobacterium *Aphanothece halophytica* under nitrogen and potassium deprivation

**DOI:** 10.3389/fbioe.2022.1028151

**Published:** 2023-01-06

**Authors:** Nattanon Chinchusak, Aran Incharoensakdi, Saranya Phunpruch

**Affiliations:** ^1^ Department of Biology, School of Science, King Mongkut’s Institute of Technology Ladkrabang, Bangkok, Thailand; ^2^ Laboratory of Cyanobacterial Biotechnology, Department of Biochemistry, Faculty of Science, Chulalongkorn University, Bangkok, Thailand; ^3^ Academy of Science, Royal Society of Thailand, Bangkok, Thailand; ^4^ Bioenergy Research Unit, School of Science, King Mongkut’s Institute of Technology Ladkrabang, Bangkok, Thailand

**Keywords:** *Aphanothece halophytica*, hydrogen production, gene expression, potassium deprivation, nitrogen deprivation

## Abstract

The unicellular halotolerant cyanobacterium *Aphanothece halophytica* is known as a potential hydrogen (H_2_) producer. This study aimed to investigate the enhancement of H_2_ production under nutrient deprivation. The results showed that nitrogen and potassium deprivation induced dark fermentative H_2_ production by *A. halophytica*, while no differences in H_2_ production were found under sulfur and phosphorus deprivation. In addition, deprivation of nitrogen and potassium resulted in the highest H_2_ production in *A. halophytica* due to the stimulation of hydrogenase activity. The effect of adaptation time under nitrogen and potassium deprivation on H_2_ production was investigated. The results showed that the highest H_2_ accumulation of 1,261.96 ± 96.99 µmol H_2_ g dry wt^−1^ and maximum hydrogenase activity of 179.39 ± 8.18 µmol H_2_ g dry wt^−1^ min^−1^ were obtained from *A. halophytica* cells adapted in the nitrogen- and potassium-deprived BG11 medium supplemented with Turk Island salt solution (BG11_0_-K) for 48 h. An increase in hydrogenase activity was attributed to the decreased O_2_ concentration in the system, due to a reduction of photosynthetic O_2_ evolution rate and a promotion of dark respiration rate. Moreover, nitrogen and potassium deprivation stimulated glycogen accumulation and decreased specific activity of pyruvate kinase. Transcriptional analysis of genes involved in H_2_ metabolism using RNA-seq confirmed the above results. Several genes involved in glycogen biosynthesis (*glgA*, *glgB*, and *glgP*) were upregulated under both nitrogen and potassium deprivation, but genes regulating enzymes in the glycolytic pathway were downregulated, especially *pyk* encoding pyruvate kinase. Interestingly, genes involved in the oxidative pentose phosphate pathway (OPP) were upregulated. Thus, OPP became the favored pathway for glycogen catabolism and the generation of reduced nicotinamide adenine dinucleotide phosphate (NADPH), which resulted in an increase in H_2_ production under dark anaerobic condition in both nitrogen- and potassium-deprived cells.

## 1 Introduction

Huge amounts of energy are used by humans in households, in industry, and in agriculture. Fossil fuel is the main energy source worldwide, but it is non-renewable and limited in supply. Due to annual increases in energy consumption, there is a high demand for fossil fuel. As a result, greenhouse gases produced from the combustion of fossil fuels have increased. This has led to environmental problems, including global warming. In addition, the amount of fossil fuel will not be sufficient for human energy consumption in the future. Hydrogen (H_2_) is an alternative energy carrier that can be used instead of the limited fossil fuel resources since its combustion releases a high heating value of 141.6 MJ kg^−1^ (Perry 1963). This combustion of H_2_ is an environmentally friendly process that does not produce greenhouse gases as by-products (Gupta and Parkhey 2017). H_2_ can be produced by various types of microorganisms, such as purple bacteria, green sulfur bacteria, green algae, and cyanobacteria.

Cyanobacteria or blue-green algae are prokaryotic and photoautotrophic microorganisms that have oxygenic photosynthetic activity (Bothe et al., 2010). Cyanobacteria can convert abundant raw materials for photosynthesis such as water, sunlight, and CO_2_ into carbohydrate and O_2_. Cyanobacteria can produce H_2_ through many processes, depending on the cyanobacterial type and metabolism. Three enzymes are involved in H_2_ metabolism in cyanobacteria, namely, bidirectional hydrogenase, uptake hydrogenase, and nitrogenase (Khetkorn et al., 2013). Despite the fact that cyanobacteria are capable of photobiological H_2_ production, all enzymes involved are sensitive to O_2_. To sustain and increase the yield of H_2_ production and prevent enzyme inactivity from O_2_, a two-stage H_2_ production scheme was proposed (Ananyev et al., 2012). In the first stage, cyanobacteria are grown in complete media to accumulate biomass. Then, cells enter the second stage by incubating under stress conditions and produce H_2_ when anaerobic conditions are established.

The deliberate depletion of nutrients in media is a technique used to induce H_2_ production and is an effective way to maintain sustainable H_2_ production in cyanobacteria (Srirangan et al., 2011). Under nitrogen deprivation, *A. halophytica* shows high H_2_ production in dark anaerobic conditions (Taikhao et al., 2013; Taikhao et al., 2015). Moreover, nitrogen deprivation also induces H_2_ evolution in *Oscillatoria brevis*, *Calothrix membranacea* (Lambert and Smith 1977), and Anabaena siamensis TISIR 8012 (Khetkorn et al., 2010; Taikhao and Phunpruch 2017). Under nitrogen deprivation, the accumulation of glycogen is carried out *via* photoautotrophic processes. The accumulated glycogen is degraded into glucose-6-phosphate with the generation of reduced nicotinamide adenine dinucleotide phosphate (NADPH), the electron donor of [NiFe]-H_2_ase, and hydrogen is generated thereafter under dark anaerobic conditions (Ananyev et al., 2008). Previous research showed that under nitrogen deprivation, the bidirectional H_2_ase activity of *A. halophytica* was obviously induced during dark anaerobic incubation and the expression of *narB* encoding ferredoxin-nitrate reductase was downregulated (Phunpruch et al., 2016). Consequently, reduced nicotinamide adenine dinucleotide (NADH) was enhanced and subsequently H_2_ was increasingly generated (Phunpruch et al., 2016). In addition, sulfur deprivation enhanced H_2_ production in *Gloeocapsa alpicola* (Antal and Lindblad 2005) and Microcystis aeruginosa (Rashid et al., 2009). Potassium deficiency increased H_2_ production by *Synechocystis* sp. PCC 6803 (Ueda et al., 2016).

The unicellular halotolerant cyanobacterium *Aphanothece halophytica*, originally isolated from Solar Lake (Israel), has the ability to grow at high salinity at concentrations of NaCl up to 3 M (Takabe et al., 1988). Since it is an obligately halophilic strain that is unable to grow under NaCl-deprived conditions, it is a model microorganism for studying the halotolerance mechanism in cyanobacteria. *A. halophytica* is well able to produce dark fermentative H_2_ under nitrogen depletion in enriched medium (Taikhao et al., 2013). It has advantages of growing and producing H_2_ when cells were incubated in natural seawater without any supplementation of NaNO_3_ (Taikhao et al., 2015). The bidirectional [NiFe]-H_2_ase was identified in *A. halophytica* (Phunpruch et al., 2016). Moreover, H_2_ production by *A. halophytica* was enhanced by cell immobilization using alginate and agar (Pansook et al., 2016; Pansook et al., 2019a). In addition, *A. halophytica* treated with photosystem II inhibitors clearly increased H_2_ production (Pansook et al., 2019b). Recently, simazine, an herbicide of the triazine class, was shown to significantly enhance H_2_ production by *A. halophytica* under dark anaerobic condition (Pansook et al., 2022). In addition, sodium sulfide was an effective reducing agent for enhancing H_2_ production by *A. halophytica* due to the reduced O_2_ concentration in the system, thus increasing hydrogenase activity (Chinchusak et al., 2022).

In this study, we focused on H_2_ production by *A. halophytica* under nutrient-deprived conditions and investigated H_2_ metabolism under these conditions. Photosynthesis and carbon and nitrogen assimilation pathways in N- and K-deprived cells were studied by RNA-seq based transcriptome analysis. H_2_ase activity, glycogen content, and pyruvate kinase activity were also determined. The patterns of the transcriptome and experimental evidence were mutually supportive.

## 2 Materials and methods

### 2.1 Cyanobacterial cultivation

Aphanothece halophytica was grown photoautotrophically in a 250-ml Erlenmeyer flask containing 100 ml of BG11 medium (pH 7.4) (Rippka et al., 1979) supplemented with Turk Island salt solution (Garlick et al., 1977). The BG11 medium consisted of 17.6 mM NaNO_3_ as a nitrogen source. The Turk Island salt solution contained high concentrations of minerals, including 0.5 M NaCl, 49 mM MgCl_2_•6H_2_O, 30 mM MgSO_4_•7H_2_O, and 8.9 mM KCl as main components. Cells were initially adjusted to a density of OD_730_ at approximately 0.1 and subsequently shaken at 120 rpm on a rotary shaker at 30°C under a white-light fluorescence intensity of 30 µmol photons m^−2^ s^−1^ with a light (18 h/day): dark (6 h/day) cycle for 7 days.

### 2.2 Cyanobacterial adaptation condition under nutrient deprivation

H_2_ production by A. halophytica was measured as part of its two-stage cultivation. Firstly, A. halophytica was grown in BG11 with Turk Island salt solution for 7 days to accumulate biomass. The seven-day-old cells were harvested by centrifugation at 8,000 × g at 4 C for 10 min before resuspension in various types of single nutrient-depleted media: nitrogen-deprived BG11 supplemented with Turk Island salt solution (BG11_0_), phosphorus-deprived BG11 supplemented with Turk Island salt solution (BG11-P), potassium-deprived BG11 supplemented with Turk Island salt solution (BG11-K), and sulfur-deprived BG11 supplemented with Turk Island salt solution (BG11-S). To remove nitrogen in BG11_0_, NaNO_3_ was omitted from BG11. In BG11-P, NaH_2_PO_4_ was omitted from BG11. To remove potassium from BG11-K, KCl was omitted from Turk Island salt solution. In addition, K_2_HPO_4_ was also omitted from BG11 and replaced by the addition of Na_2_HPO_4_. In BG11-S, ZnSO_4_•7H_2_O and CuSO_4_•5H_2_O were omitted from BG11 and replaced by ZnCl_2_ and CuCl_2_, respectively. In addition, MgSO_4_•7H_2_O was omitted from both BG11 and Turk Island salt solution and replaced by the addition of MgCl_2_. Thereafter, the cell suspensions were incubated for 24 h before H_2_ was measured, under anaerobic conditions.

### 2.3 H_2_ measurement

Aphanothece halophytica adapted in each single nutrient-deprived media for 24 h was harvested by centrifugation and resuspended in a fresh medium. Five ml of cell suspension was transferred to a 10-ml glass vial. The vial was sealed with a rubber stopper and the cyanobacterial cells were made to enter anaerobic conditions by purging with argon gas for 10 min. Each cell suspension was then incubated at 30°C under darkness for 2 h before H_2_ measurement was undertaken. H_2_ measurement was performed using a Gas Chromatograph (Hewlett-Packard HP5890A, Japan) with a molecular sieve 5 °A 60/80 mesh packed column and a thermal conductivity detector using argon as the carrier gas (Baebprasert et al., 2010). H_2_ production was expressed as µmol H_2_ evolved per milligram dry cell weight. All experiments were done in triplicate.

### 2.4 Hydrogenase activity measurement

Bidirectional hydrogenase activity was determined by measuring H_2_ evolution in the presence of dithionite-reduced methyl viologen. The reaction mixture comprised 1 ml of cell suspension and 1 ml of 25 mM sodium phosphate buffer (pH 7.6) containing 10 mM methyl viologen dichloride hydrate (Roche, Germany) and 40 mM sodium dithionite (Sigma, Germany). The mixture was incubated under argon atmospheric conditions at 30°C under darkness for 30 min. H_2_ from headspace was subsequently analyzed by a gas chromatograph.

### 2.5 Dry cell weight and glycogen content determination

For each dry cell weight determination, 5 ml of cyanobacterial cell suspension was filtered through a glass microfiber filter GF/C (Whatman, United Kingdom). Sterile distilled water was used to wash the cells collected on the microfiber filter. Then, the filter containing cells was dried in an oven at 70°C until weight was constant. The dry cell weight was calculated. For glycogen content determination, glycogen extraction and hydrolysis were performed following the previous studied procedure (Ernst et al., 1984). *A. halophytica* cells that had been adapted in various media were harvested by centrifugation at 8,000 × g at 4°C for 10 min and resuspended in fresh medium that contained chlorophyll *a* at a concentration of 300 µg chl *a* mL^−1^. Fifty µL of each cell suspension was added to 200 µL of 30% (w/v) KOH. The mixture was then incubated at 100 °C in a water bath for 90 min. Cells were lysed by ultrasonication at 20% pulse for 5 min on ice. To precipitate glycogen, 600 µL of absolute ethanol was added into the extract. Each sample was kept on ice for 1 h. Glycogen was collected by centrifugation at 12,000 × g for 5 min at 4 C. Then, each glycogen pellet was washed twice by absolute ethanol and subsequently dried at 60°C for 10 min. Glycogen was resuspended in 300 µL of 100 mM acetate buffer (pH 4.75). For each sample, glycogen was digested into glucose by adding amyloglucosidase from *Aspergillus niger* (Sigma, United States) and α-amylase from *Aspergillus oryzae* (Sigma, Switzerland) at final concentrations of 4 and 8 units, respectively. The reaction was incubated at 25°C for 1 h. Insoluble membrane fragments were removed by centrifugation at 12,000 × g at 4°C for 5 min. Total sugar in each supernatant was determined by phenol-sulfuric acid assay (Dubois et al., 1956). Standard glycogen from bovine liver (Sigma, United States) (0–100 µg) was used. The glycogen content was calculated using a standard calibration curve. Each experiment was performed in triplicate.

### 2.6 Pyruvate kinase activity determination

Pyruvate kinase activity was determined indirectly by measuring the oxidation of NADH in the reaction catalyzed by lactate hydrogenase. The activity of pyruvate kinase was determined by a protocol previously described (Malcovati and Valentini, 1982). To prepare cell-free extract of *A. halophytica*, 100 ml of *A. halophytica* cell culture was harvested by centrifugation at 8,000 × g at 4°C for 10 min and subsequently resuspended in 1 ml of 10 mM (2-[4-(2-hydroxyethyl) piperazine-1-yl] ethanesulfonic acid (HEPES) buffer (pH 7.5). Cells were lysed using 20% pulse of ultrasonication on ice for 5 min. The cell-free extract was obtained after centrifugation at 8,000 × g at 4°C for 10 min. To assay pyruvate kinase activity, 50 µL of cell-free extract was added into 950 µL of reaction mixture containing 10 mM HEPES buffer, 10 mM MgCl_2_, 50 mM KCl, 20 mM ADP (Adenosine 5′-diphosphate sodium salt) (Sigma, United States), 10 mM PEP (Phosphoenolpyruvic acid monopotassium salt) (Sigma, Switzerland), 5 mM NADH (β-nicotinamide adenine dinucleotide, reduced disodium salt hydrate) (Sigma, United States), and 0.5 U of L-lactate dehydrogenase (Roche, Germany). The reaction mixture without PEP was used as a negative control. The decrease of NADH was measured spectrophotometrically at a wavelength of 340 nm at 25°C. One unit of pyruvate kinase activity is defined as the amount of enzyme that catalyzes the production of 1.0 µmol pyruvate from the substrate PEP in 1 min. The specific activity of pyruvate kinase was measured as activity per total protein concentration. Protein concentration was determined by Bradford assay (Bradford 1976).

### 2.7 mRNA sequencing by Illumina HiSeq/Novaseq or MGI2000

Seven-day old *A. halophytica* cells adapted in BG11_0_, BG11-K, and BG11_0_K for 2 days were harvested by centrifugation at 12,000 × g at 4 C for 10 min. The total RNA of each sample was extracted using QIAzol Lysis Reagent (Qiagen, United States). Then, total RNA was qualified and quantified using a NanoDrop 2000/2000c spectrophotometer (Thermo Fisher Scientific, United States). Ribosomal RNA (rRNA) was removed by using a Ribo-Zero rRNA removal Kit (Epicentre, United States) following the manufacturer’s instructions. The construction of the next generation sequencing library was conducted by Genewiz (China) according to the standard protocols. The different indices of libraries were multiplexed and loaded on an Illumina HiSeq/Novaseq instrument according to the instructions of the manufacturer (Illumina, United States). RNA sequencing was performed using the 2 × 150 paired end (PE) configuration. Image analysis and base calling were conducted using the HiSeq Control Software (HCS) + OLB + GAPipeline-1.6 (Illumina) on the HiSeq instrument. All RNA sequencing and alignment processes were conducted by Genewiz (China).

### 2.8 Statistical analysis

All experiments were performed in triplicate. All data are shown as the mean ± standard deviation. One-way analysis of variance (ANOVA) was used for analysis to the 95% confidence level with IBM SPSS version 19 software.

## 3 Results

### 3.1 H_2_ production in deprived and limited media

**FIGURE 1:**
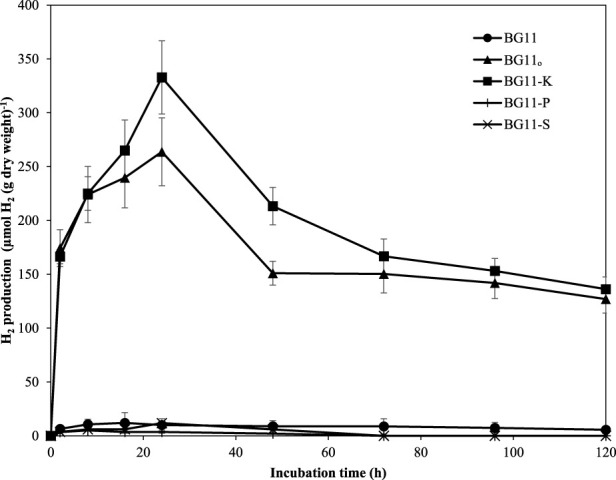
H_2_ production (µmol H_2_ g dry wt^−1^) by *A. halophytica* incubated for various times in BG11 (•), BG11_0_ (▲), BG11-K (■), BG11-P (+), and BG11-S (*) medium under dark anaerobic condition. Data are means ± SD (*n* = 3).

The result showed that *A. halophytica* cells incubated in BG11_0_ and BG11-K showed clearly higher H_2_ production than those in BG11, BG11-P, and BG11-S (Figure 1). The highest H_2_ accumulation of 332.82 ± 34.02 µmol H_2_ g dry cell wt^−1^ was shown by cells incubated in BG11-K for 24 h. It was 33-fold higher than that of cells cultivated in normal BG11 medium (10.01 ± 5.92 µmol H_2_ g dry cell wt^−1^) (Figure 1). In addition, cells incubated in BG11_0_ accumulated maximum H_2_ of 263.75 ± 31.58 µmol H_2_ g dry cell wt^−1^, or 26-fold higher than that of cells incubated in normal BG11 medium (Figure 1). This study demonstrated the activation of H_2_ production by *A. halophytica* under nitrogen and potassium deprivation.

In this study, the effects of various concentrations of nitrogen and potassium on H_2_ production and H_2_ase activity by *A. halophytica* with focus on potassium and nitrogen deprivation was investigated. The results showed that during potassium deprivation, a decrease in nitrogen concentration resulted in higher H_2_ase activity and H_2_ production by *A. halophytica* (Table 1). Similarly, during nitrogen deprivation, lower potassium concentration resulted in significantly higher H_2_ production and H_2_ase activity by *A. halophytica* (Table 1)*.* The highest H_2_ase activity with 120.05 ± 8.98 µmol H_2_ g dry wt^−1^ min^−1^ and maximum H_2_ accumulation with 507.51 ± 13.78 µmol H_2_ g dry wt^−1^ was shown in cells incubated under a combined K- and N-deprived conditions (Table 1). This H_2_ase activity and H_2_ production was approximately 40 and 50-fold higher than those under normal conditions (incubated in BG11), respectively. The deprivation of both nitrogen and potassium together promoted H_2_ase activity, and thus resulted in higher H_2_ production.

**TABLE 1:** H_2_ production and H_2_ase activity by *A. halophytica* cells incubated in various types of media: BG11 supplemented with Turk Island salt solution (BG11), potassium-deprived BG11 supplemented with Turk Island salt solution (BG11-K) containing various concentrations of NaNO_3_, nitrogen-deprived BG11 supplemented with Turk Island salt solution (BG11_0_) containing various concentrations of KCl, and potassium- and nitrogen-deprived BG11 supplemented with Turk Island salt solution (BG11_0_-K) for 24 h. Data are means ± SD (*n* = 3). Different letters in columns indicate a significant difference, and the same letter indicates no significant difference according to Duncan’s multiple range test at *p* < 0.05.

Type of media	KCl (mM)	NaNO_3_ (mM)	H_2_ production (µmol H_2_ g dry weight^−1^)	H_2_ase activity (µmol H_2_ g dry weight^−1^ min^−1^)
BG11	8.93	17.6	10.55 ± 0.58^h^	3.20 ± 0.22^f^
BG11-K	0	17.6	332.58 ± 8.56^ef^	51.38 ± 1.51^de^
	0	13.2	354.44 ± 9.15^de^	60.30 ± 4.68^cd^
	0	8.8	379.38 ± 17.51^cd^	62.97 ± 4.78^c^
	0	4.4	449.32 ± 6.08^b^	81.23 ± 7.03^b^
BG11_0_	8.93	0	260.34 ± 12.24^g^	46.61 ± 3.30^e^
	6.70	0	279.10 ± 17.43^g^	51.83 ± 1.40^de^
	4.47	0	313.99 ± 21.93^f^	54.9 ± 3.10^cde^
	2.23	0	388.94 ± 34.66^c^	84.70 ± 6.65^b^
BG11_0_-K	0	0	507.51 ± 13.78^a^	120.05 ± 8.98^a^

### 3.2 Time course of H_2_ase activity, H_2_ production, and O_2_ production under nitrogen and potassium deprivation

**FIGURE 2:**
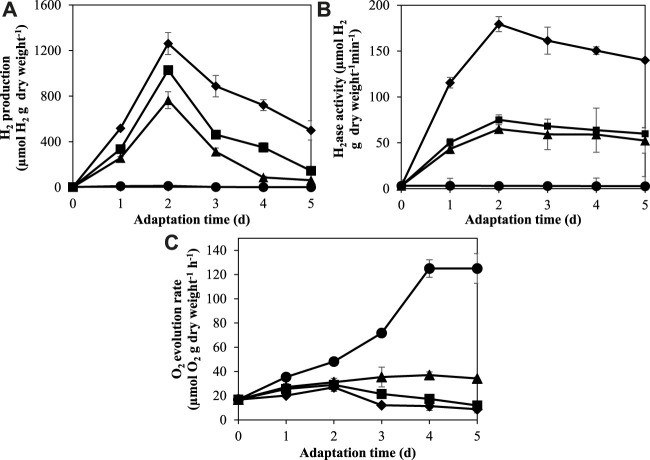
Effect of adaptation time on H_2_ production **(A)**, H_2_ase activity **(B)**, and O_2_ evolution rate **(C)** by *A. halophytica* cells. *A. halophytica* was adapted in various kinds of media for various times, BG11 (•), BG11_0_ (▲), BG11-K (■), and BG11_0_-K (⬧) for 1–5 days before harvesting and resuspending in a fresh medium. H_2_ production was measured in incubated cells under dark anaerobic condition for 24 h. Data are means ± SD (*n* = 3).

In this study, *A. halophytica* cells were adapted in BG11, BG11_0_, BG11-K, and BG11_0_-K media under photoautotrophic conditions with light exposure (18 h/day) for 1, 2, 3, 4, and 5 days. The hydrogenase activity, H_2_ production, and O_2_ production of adapted cells were measured after incubation under dark anaerobic condition for 24 h. The result showed that under nitrogen and/or potassium deprivation, *A. halophytica* cells gave higher H_2_ production and hydrogenase activity than cells under normal condition (Figures 2A, B). On the other hand, O_2_ production was highest in cells adapted in normal BG11 medium (Figure 2C). The combined deprivation of nitrogen and potassium promoted higher H_2_ase activity and H_2_ production in *A. halophytica* than did single nutrient deprivation. *A. halophytica* incubated under nitrogen and potassium deprivation showed maximum H_2_ production rate of 40.87 ± 1.07 µmol H_2_ g dry wt^−1^ h^−1^. The highest H_2_ production of 1,261.96 ± 96.99 µmol H_2_ g dry wt^−1^ was obtained in cells adapted in BG11_0_-K for 2 days (Figure 2A). The lowest O_2_ evolution rate of 8.81 ± 2.93 nmol O_2_ g dry wt^−1^ h^−1^ was obtained in cells adapted in BG11_0_-K after 3 days (Figure 2C). It was found that H_2_ production was related to H_2_ase activity (Figures 2A, B). H_2_ase activity increased dramatically at the first 48 h of adaptation incubation and the highest H_2_ase activity with 179.39 ± 8.18 µmol H_2_ g dry wt^−1^ min^−1^ was found in cells adapted in BG11_0_-K for 2 days (Figure 2B).

### 3.3 Glycogen accumulation under nitrogen and potassium deprivation

**FIGURE 3:**
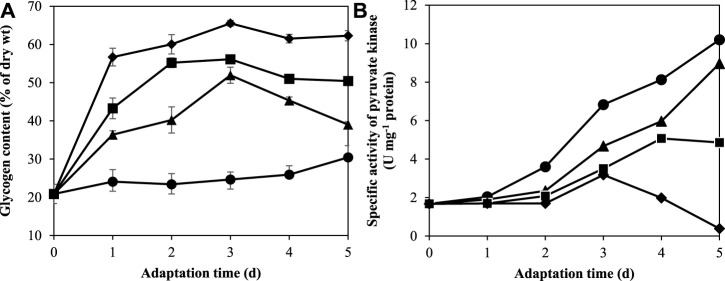
Effect of adaptation time on glycogen content **(A)** and specific activity of pyruvate kinase **(B)** by *A. halophytica* cells. *A. halophytica* was adapted in various kinds of media for various times, BG11 (•), BG11_0_ (▲), BG11-K (■), and BG11_0_-K (⬧) for 1–5 days before harvesting and resuspending in a fresh medium. H_2_ production was measured in incubated cells under dark anaerobic condition for 24 h. Data are means ± SD (*n* = 3).

The glycogen content of *A. halophytica* cells adapted in BG11, BG11_0_, BG11-K, and BG11_0_-K media for 1, 2, 3, 4, and 5 days was measured. The results revealed that under nitrogen and/or potassium deprivation, *A. halophytica* clearly produced and accumulated higher intracellular glycogen than under normal conditions. The glycogen content of cells adapted in BG11_0_, BG11-K, and BG11_0_-K clearly increased during the first 3 days of adaptation, whereas in the case of BG11 adapted cells, it was almost constant over the period of adaptation (Figure 3A). The highest glycogen content at 65.53 ± 0.78% of cell dry wt was obtained in cells adapted in BG11_0_-K for 3 days (Figure 3A). It was approximately three times higher than glycogen content of cells adapted in BG11 (24.66 ± 1.97% of cell dry wt) (Figure 3A). In the case of only single-nutrient deprivation, glycogen accumulated under potassium deprivation was higher than that under nitrogen deprivation. Under K-deprivation, cells accumulated the maximum glycogen content of 56.13 ± 0.66% of cell dry wt whereas under N-deprivation, cells accumulated the maximum glycogen content at 51.95 ± 2.11% of cell dry wt after 3 days of adaptation.

### 3.4 Specific activity of pyruvate kinase under nitrogen and potassium deprivation

Pyruvate kinase (PK) is known to be activated by K^+^ (Oria-Hernández et al., 2005). It has an important role in regulating the glycolytic pathway in carbohydrate metabolism. The measurement of pyruvate kinase (PK) activity was performed in *A. halophytica* cells adapted in BG11, BG11_0_, BG11-K, and BG11_0_-K for 1, 2, 3, 4, and 5 days. The results showed that specific activity of PK was highest with 10.20 ± 0.60 U mg^−1^ protein in *A. halophytica* adapted in BG11 for 5 days (Figure 3B). The deprivation of either nitrogen or potassium reduced specific activity of PK significantly compared with normal conditions (Figure 3B). Similarly, combined deprivation of both nitrogen and potassium had significant effects on PK activity. It was especially notable that after 5 days of adaptation time, cells clearly showed very low PK specific activity at 0.39 ± 0.01 U mg^−1^ protein (Figure 3B).

### 3.5 Photosynthetic oxygen evolution rate and respiration rate under nitrogen and potassium deprivation

Since O_2_ is a strong inhibitor of H_2_ase, the presence of O_2_ in systems plays an important role in H_2_ production. In cyanobacteria, O_2_ is generated *via* oxygenic photosynthesis and the produced O_2_ is consumed in cellular respiration *via* the electron transport chain. To investigate how O_2_ evolution decreased under nitrogen and potassium deprivation, the photosynthetic O_2_ evolution rate and respiration rate of *A. halophytica* cells adapted in BG11, BG11_0_, BG11-K, and BG11_0_-K for 2 days was measured. The results showed that the highest photosynthetic O_2_ evolution rate of 16.94 ± 0.64 nmol O_2_ mg dry wt^−1^ min^−1^ was found in cells adapted in BG11. Under single or combined nitrogen and potassium deprivation, its photosynthetic O_2_ evolution rate decreased (Table 2). The combined deprivation of both nitrogen and potassium resulted in the lowest photosynthetic O_2_ evolution rate of 2.78 ± 0.09 nmol O_2_ mg dry wt^−1^ min^−1^ (Table 2). On the contrary, O_2_ consumption by respiration increased under nitrogen and/or potassium deprivation (Table 2). The ratio of photosynthetic O_2_ evolution rate and respiratory rate was calculated and evaluated. This ratio implies the quantity of O_2_ in a vial container. The highest ratio between photosynthetic O_2_ evolution rate and respiratory rate of 22.49 ± 0.81 was found in cells adapted in BG11 for 24 h whereas the lowest ratio of 2.57 ± 0.04 was shown in cells adapted in BG11_0_-K for 24 h (Table 2).

**TABLE 2:** Photosynthetic O_2_ evolution rate, dark respiration rate, and ratio of photosynthetic O_2_ evolution rate and respiratory rate in A. halophytica cells incubated in BG11, BG110, BG11-K, and BG110-K under dark anaerobic condition for 24 h before measuring. Data are means ± SD (*n* = 3). Different letters in columns indicate a significant difference, and the same letter indicates no significant difference according to Duncan’s multiple range test at *p* < 0.05.

Type of media	Photosynthetic O_2_ evolution rate (nmol O_2_ mg dry wt^−1^ h^−1^)	Dark respiration rate (nmol O_2_ mg dry wt^−1^ h^−1^)	Ratio of photosynthetic O_2_ evolution rate and dark respiration rate
BG11	16.94 ± 0.64^a^	0.75 ± 0.03^c^	22.49 ± 0.81^a^
BG11_0_	5.39 ± 0.87^b^	1.12 ± 0.01^b^	4.79 ± 0.73^b^
BG11-K	5.47 ± 0.59^b^	1.31 ± 0.04^a^	4.17 ± 0.57^b^
BG11_0_-K	2.78 ± 0.09^c^	1.08 ± 0.02^b^	2.57 ± 0.04^c^

### 3.6 RNA-seq based transcriptome analysis of genes involved in photosynthetic, carbon, and nitrogen assimilation pathways under nitrogen and potassium deprivation

**FIGURE 4:**
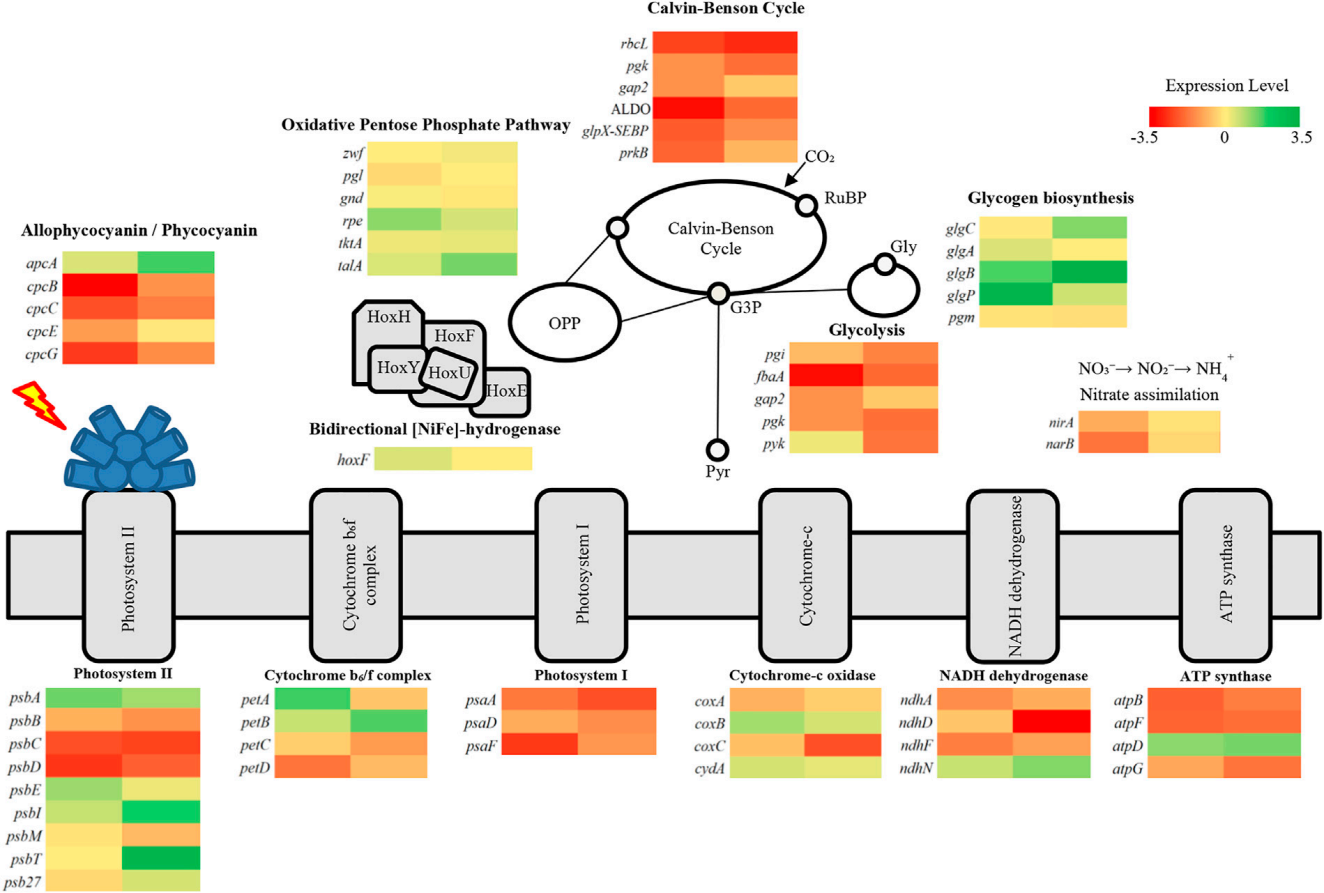
Transcriptional level of genes involved in photosynthetic pathway, carbon assimilation such as Calvin-Benson cycle, glycolysis, oxidative pentose phosphate pathway, and nitrate assimilation in *A. halophytica.* The first column shows differences of gene expression between N-deprived adapted cells and normal cells and the second column shows differences of gene expression between K-deprived adapted cells and normal cells. Expression levels are shown as heat maps with gene names, demonstrating different gene expression in many metabolic pathways involved in H_2_ production. The metabolic pathways are photosynthesis, oxidative pentose phosphate, glycolytic pathway, glycogen biosynthesis, and nitrate assimilation. Abbreviations: oxidative pentose phosphate pathway, OPP; glucose-3-phosphate, G3P; glycogen, gly; pyruvate, pyr; [NiFe]-bidirectional H_2_ase enzyme subunits, HoxEFUYH.

Gene expression based on the RNA-seq of *A. halophytica* under nitrogen and potassium deprivation was compared with that under normal conditions. The results are shown in Figure 4. Transcripts of genes involved in photosynthetic, carbon, and nitrogen assimilation pathways were analyzed. In this study, log_2_FoldChange (log_2_FC) was used to indicate the differences between transcript expression in the experimental and control groups. The results showed that genes encoding allophycocyanin and phycobilisome under N and K deprivation were expressed as log_2_FC ≤ -0.1. However, ApcA, the allophycocyanin alpha subunit encoded by *apcA*, was upregulated by 0.5 and 1.77 of log_2_FC under N and K deprivation, respectively. In addition, the expression of different genes involved in photosynthesis showed harmonic direction under N and K deprivation. Most of these genes were upregulated by log_2_FC ≤ 0.54. Especially, D1 protein encoded by *psbA* was upregulated by log_2_FC of 1.48 and 1.03 under N and K deprivation, respectively. However, the CP47, CP43 chlorophyll apoproteins, and P_680_ reaction center D2 protein encoded by *psbB*, *psbC*, and *psbD* respectively were downregulated.

Under N and K deprivation, genes involved in the Calvin-Basham-Benson (CBB) cycle were downregulated (Figure 4). Ribulose-1,5-bisphosphate carboxylase/oxygenase large subunit encoded by *rbcL* was significantly down-regulated by log_2_FC of −2.46 and −1.83 under N and K deprivation, respectively. All genes in this pathway were differently expressed by log_2_FC ≤ −0.53. Likewise, several genes involved in glycolysis were downregulated under these starvation conditions. However, pyruvate kinase encoded by *pyk* was expressed differentially, as log_2_FC of 0.26 and −1.77 under N and K deprivation, respectively. Interestingly, most genes involved in glycogen biosynthesis were upregulated. For example, 1,4-alpha-glucan branching enzyme encoded by *glgB* was upregulated as log_2_FC of 1.69 and 4.37 under N and K deprivation, respectively. In the oxidative pentose phosphate pathway (OPP), some genes were upregulated under N and K deprivation. For example, *rpe* encoding ribulose phosphate 3-epimerase was upregulated by 1.23 and 0.52 of log_2_FC, respectively. In terms of nitrogen assimilation, as expected, ferredoxin-nitrite reductase encoded by *nirA* and *nirB* was downregulated as log_2_FC of −0.94 and −1.76, respectively, under N-deprived conditions. On the contrary, no differences of expression of these genes were found under K-deprived conditions.

## 4 Discussion

Cyanobacteria are oxygenic microorganisms that can produce H_2_ under dark anaerobic conditions, especially when they are incubated in nutrient-deprived media (Ananyev et al., 2008; Baebprasert et al., 2010; Min and Sherman 2010). In a previous study, the unicellular halotolerant cyanobacterium *A. halophytica* showed the ability to produce H_2_ under nitrogen starvation (Taikhao et al., 2013; Taikhao et al., 2015; Phunpruch et al., 2016). Previously, H_2_ production was shown to be catalyzed by bidirectional [NiFe]-H_2_ase (Phunpruch et al., 2016). The results in this study demonstrated that, apart from nitrogen starvation, potassium deprivation could also significantly enhance H_2_ production by *A. halophytica* under dark anaerobic conditions compared with normal conditions (Figure 1). Potassium and nitrogen deprivation could induce glycogen catabolism with subsequent generation of increased electrons and hydrogenase activity was simultaneously induced after entering anaerobic conditions. After 24 h of incubation, H_2_ production was decreased, as a result of the decreased number of electrons and the lower hydrogenase activity. Under potassium and nitrogen deprivation, biomass was not much changed due to the low level of nutrient concentration for cell division. However, *A. halophytica* incubated in seawater with the supplementation of 378 mmol C L^−1^ glucose, 0.25 M NaCl, and 0.4 µM Fe^3+^ at 35°C, pH six gave the highest H_2_ production at day 8 of dark incubation under anoxic condition, and the high yield of H_2_ was sustained at least up to 14 days (Taikhao et al., 2015).

Under potassium deprivations, a limitation of the NaNO_3_ concentration available in BG11 medium induced both H_2_ase activity and H_2_ production (Table 1). Similarly, under nitrogen deprivations, a limited KCl concentration in BG11 medium induced both H_2_ase activity and H_2_ production (Table 1). In a previous study, it was shown that the presence of potassium reduced H_2_ase activity in *Synechocystis* 6,803, under dark anaerobic conditions, which might have been due to competitive utilization of limited NADPH for H_2_ and organic acid production (Ueda et al., 2016). Moreover, it has been suggested that potassium deprivation decreased PSII activity, lowering the production of O_2,_ which is an inhibitor of H_2_ase enzyme. Furthermore, potassium deprivation increased the degradation of starch in green alga *Scenedesmus obliquus* (Papazi et al., 2014) and in *Tetraspora* sp. CU2551 (Pewnual et al., 2022). With nitrogen deprivation, H_2_ase activity increased in *Anabaena siamensis* TISTR8012, resulting in enhanced H_2_ production (Taikhao and Phunpruch 2017). On the other hand, phosphorus and sulfur deprivation did not promote H_2_ production by *A. halophytica.* In addition, potassium, sulfur, and phosphate deprivation was observed to likely have important effects on nucleic acids, protein synthesis, and other metabolic pathways in cells, such as photosynthesis, cellular respiration, and H_2_ metabolism (Warichanan and Phunpruch 2019). Moreover, a combination of nitrogen and sulfur depletion enhanced H_2_ production in *Synechocystis* sp. strain PCC 6803 (Baebprasert et al., 2010) and in *Arthrospira* sp. PCC 8005 (Raksajit et al., 2012). Table 3 shows the comparison of maximum H_2_ production rate by *A. halophytica* under various conditions. It was shown that deprivation of potassium together with nitrogen promoted higher H_2_ production by *A. halophytica* than only nitrogen deprivation. However, H_2_ production rate by free cells of *A. halophytica* in this study was lower than that of agar-immobilized cells (Pansook et al., 2019b) and free cells incubated in BG11_0_ treated with 25 µM simazine (Pansook et al., 2022) and 50 mM sodium sulfide (Chinchusak et al., 2022).

**TABLE 3:** Maximum dark fermentative H_2_ production rate by *A. halophytica* incubated under various conditions.

Condition	Maximum H_2_ production rate	References
Free cells incubated in BG11_0_-K, dark, 120 rpm, pH 7.4, 30°C	40.87 ± 1.07 µmol H_2_ g dry wt^−1^ h^−1^	This study
Free cells incubated in BG11_0_ with 0.4 μM Fe^3+^, dark, 120 rpm, pH 7.4, 30°C	13.80 ± 0.373 μmol H_2_ mg^−1^ chl a h^−1^	Taikhao et al. (2013)
Free cells incubated in optimal seawater, dark, 120 rpm, pH 6.0, 35°C	82.79 ± 3.47 μmol H_2_ g dry wt^−1^ h^−1^	Taikhao et al. (2015)
Alginate-immobilized cells incubated in BG11_0_, 120 rpm, pH 7.4, 30°C	0.532 µmol H_2_ mg chl a^−1^ h^−1^	Pansook et al. (2016)
Free cells incubated in BG11_0_ with 0.5 µM CCCP, dark, 120 rpm, pH 7.4, 30°C	39.50 ± 2.13 µmol H_2_ g dry wt^−1^ h^−1^	Pansook et al. (2019a)
Agar-immobilized cells incubated in BG11_0_, dark, 120 rpm, pH 7.4, 40°C	135.54 ± 1.92 μmol H_2_ g dry wt^−1^ h^−1^	Pansook et al. (2019b)
Free cells incubated in BG11_0_ with 25 µM simazine, dark, 120 rpm, pH 7.4, 30°C	58.88 ± 0.22 µmol H_2_ g dry wt^−1^ h^−1^	Pansook et al. (2022)
Free cells incubated in BG11_0_ with 50 mM sodium sulfide, dark, 120 rpm, pH 7.4, 30°C	542.45 ± 35.99 µmol H_2_ g dry wt^−1^ h^−1^	Chinchusak et al. (2022)

H_2_ase, including the [NiFe]-H_2_ase enzyme in cyanobacteria, is usually sensitive to the presence of O_2_ (McIntosh et al., 2011). The fundamental pathways involved in O_2_ generation and consumption in cyanobacteria are photosynthesis and dark respiration, respectively. Our result showed that a combined deprivation of both nitrogen and potassium promoted H_2_ production by reducing O_2_ concentration in the vial (Figure 2). A decrease in O_2_ concentration induced H_2_ase activity, resulting in a higher H_2_ production (Figure 2). The balance of O_2_ concentration in a system therefore plays a decisive role in H_2_ production. In cyanobacteria, O_2_ in the system is normally generated by photosynthetic activity of PSII. However, a measurement of H_2_ production in this study was performed under darkness when photosynthetic activity should have been less than under light conditions. The deprivation of either nitrogen or potassium reduced the photosynthetic O_2_ evolution rate by 3-fold, whereas combined deprivation of both nitrogen and potassium reduced the photosynthetic O_2_ evolution rate by 6-fold (Table 2). A decrease in photosynthetic O_2_ evolution rate caused the reduction of O_2_ in the system. This in turn induced H_2_ase activity, resulting in higher H_2_ production. In the case of nitrogen deprivation, the result was in agreement with a previous study showing the reduction of photosynthetic activities in *Synechococcus elongatus* PCC 7942 under nitrogen starvation (Choi et al., 2016). Potassium deprivation decreased photosynthetic pigments and activity in *Synechocystis* sp. strain PCC 6803 (Nanatani et al., 2015) and in *Anabaena torulosa* (Alahari and Apte 1998).

In addition, the produced O_2_ can be consumed by dark intracellular respiration. An increased respiration rate for *A. halophytica* was observed in cells deprived of nitrogen or potassium or both (Table 2). As a result, a decreased level of O_2_ in the vial containing cells incubated under nitrogen and potassium deprivation was obtained. The ratio of photosynthetic O_2_ evolution rate and dark respiration rate was used to monitor the level of O_2_ concentration in the system (Table 2). A higher ratio indicated higher activity of photosynthetic O_2_ evolution activity over respiration activity, suggesting the presence of high O_2_ concentration in the vial, and thus lower H_2_ase activity and lower H_2_ production. A low ratio of photosynthetic O_2_ evolution rate to dark respiration rate was found in nitrogen- and potassium-free adapted cells (Table 2). This probably involved a combined effect of nitrogen and potassium deprivation suppressing related genes. In terms of photosynthetic O_2_ evolution: respiratory rate ratio (Table 2), the results implied that the lower the ratio, the lower the O_2_ concentration. Cells incubated in deprived media showed lower photosynthetic O_2_ evolution rate and dark respiration rate than did normal cells. Several genes encoding cytochrome *c* oxidase and NADH dehydrogenase were upregulated in cells under both nitrogen and potassium starvation (Figure 4). The results suggested that *A. halophytica* increased dark respiration under nitrogen and potassium starvation. However, no significant differences of expression of genes encoding respiratory electron transport system were reported in *Synechococcus elongatus* PCC 7942 under nitrogen deprivation (Choi et al., 2016).

H_2_ production by *A. halophytica* was established by the two-stage regime: cyanobacterial growth followed by adaptation in nutrient deprivation condition to induce an increase of H_2_ production. Firstly, cells were grown in rich BG11 medium for 7 days to accumulate biomass. Then, the cells were made to enter the adaptation period, or the second stage, by incubation in nutrient-deprived media to accumulate glycogen under illumination and aerobic conditions. Subsequently, cells were transferred into a glass vial and H_2_ production was induced under dark anaerobic condition. The adaptation period during nutrient deficiency was very crucial since cells incubated in deprived media needed to accumulate a high content of glycogen, or other chemical compounds, in order to provide electrons for H_2_ase to produce H_2_. Individually and combined nitrogen and potassium deprivation were chosen for this study. The result showed that H_2_ase activity and H_2_ production was highest in cells adapted in nitrogen- and potassium-deprived media for 48 h (Figures 2A, B), suggesting a high accumulation of glycogen in adapted cells. Glycogen in the adapted cells was extracted and determined. Glycogen was seen to have accumulated more in cells incubated in nitrogen- and potassium-deprived media compared to that of cells incubated in BG11 (Figure 3B). The highest glycogen was found in combined nitrogen- and potassium-free cells during the adaptation period (Figure 3B). This result was confirmed with transcriptional analysis. Transcriptional analysis by RNA-seq showed that the *glgA* and *glgB* genes encoding the starch synthase and 1,4-alpha-glucan branching enzymes, respectively, were upregulated under both nitrogen and potassium deprivation (Figure 4). The results suggested that a lack of nitrogen and/or potassium causes an increase in glycogen content, giving rise to higher H_2_ production (Taikhao et al., 2015).

Under dark anaerobic condition, cyanobacterial H_2_ can be produced *via* [NiFe] H_2_ase reduced by NAD(P)H (Gutekunst et al., 2014) and reduced ferredoxin (Meuer et al., 1999; Gutekunst et al., 2014). The main sources of reductants for H_2_ generation under dark anaerobic condition are NADH and reduced ferredoxin from glycolysis and NADPH from catabolism of accumulated glycogen, provided by oxidative pentose phosphate (OPP) pathway (Kumaraswamy et al., 2013). In this study, the specific activity of PK in glycolytic pathway decreased in *A. halophytica* under K deprivation (Figure 3A). The specific activity of PK in K- and NK- free adapted cells was less than that in abundant and N-free adapted cells throughout the period of adaptation time. PK is known to be induced by K^+^ and it is less activated when K is absent in a system (Oria-Hernández et al., 2005). Therefore, this associated enzyme regulation might have controlled the flux of glycogen catabolism in *A. halophytica*. Furthermore, GAPDH-2 has a major role in the CBB cycle, using NADPH as a preferred electron donor to reduce 1,3-bis-phosphoglycerate to GAP (Koksharova et al., 1998). Under potassium deprivation, transcriptional analysis revealed that *pyk* and *gap2* encoding PK and GAPDH-2, respectively, were downregulated (Figure 4). Consequently, in potassium-free adapted cells, it could be suggested that upper-glycolytic metabolites could be in excess, especially glucose-6-phophate (G6P). G6P is a broken-down molecule of glycogen, and the branching point between glycolysis and OPP pathway. For a reduced activity of both PK and GADPH-2, it could be the case that G6P preferentially enters the OPP pathway, generating more NADPH and using less NADPH in CBB and glycolysis. Therefore, OPP pathway may well be the favored pathway for H_2_ production in potassium-deprived adapted cells of *A. halophytica* under dark anaerobic conditions. In a previous study, the *pykF* knocking out *Escherichia coli* mutant provided higher activity of enzymes in OPP, but the activities of glycolytic enzymes decreased compared with those in wild-type cells (Siddiquee et al., 2004). Moreover, low activity of pyruvate kinase was shown to promote respiration in yeast (Grüning et al., 2011). Prior to this study, H_2_ production was induced in nitrogen-free cells because the expression of genes involved in glycogen catabolism had increased (Osanai et al., 2006). Moreover, several genes in the OPP pathway were extremely upregulated in *Synechocystis* sp. PCC 6803 incubated in nitrogen-free medium (Osanai et al., 2006). This was probably due to G6P being degraded through the OPP pathway. It seems that glycogen catabolism might preferentially take place *via* the OPP pathway, producing more NAD(P)H compared to that *via* the glycolytic pathway in cells incubated in both nitrogen- and potassium-deprived conditions. Therefore, the described effects of both nitrogen and potassium deprivation on H_2_ production may have been synergistic, and accelerated H_2_ production in *A. halophytica* under dark anaerobic conditions.

**FIGURE 5:**
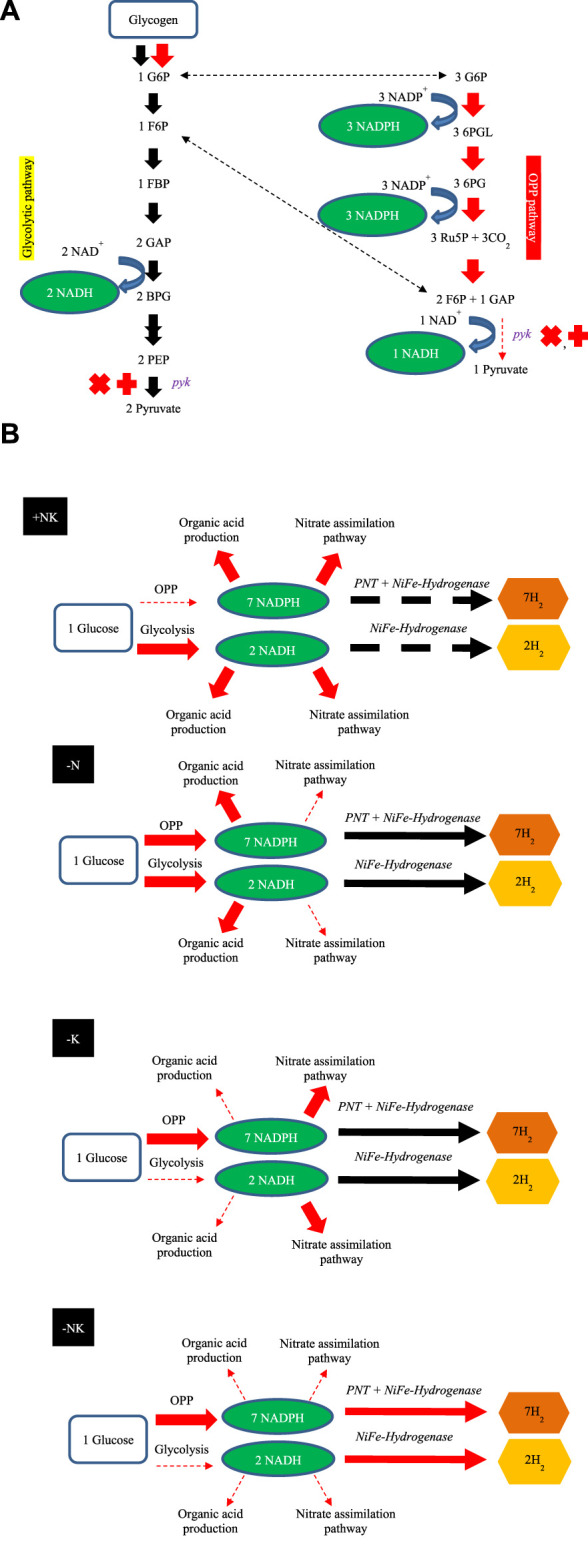
Proposed schematic mechanism of H_2_ production by *A. halophytica* based on the RNA-seq after adaptation in nitrogen and/or potassium deprivation for 2 days. Glycolytic and OPP pathways are involved in NAD(P)H generation *via* glycogen catabolism **(A)**. Proposed H_2_ metabolism and involved pathways of *A. halophytica* incubated in BG11 (+NK), BG11_0_ (-N), BG11-K (-K), and BG11_0_-K (-NK) under dark anaerobic conditions **(B)**. Arrows show electron transport direction in each pathway.

The schematic H_2_ production by *A. halophytica* during deprivation of individual nitrogen and potassium, and combined nitrogen and potassium, under dark anaerobic conditions is shown in Figure 5. The reductants involved in H_2_ metabolism by cyanobacteria, NADH, and NADPH are generated mainly by the glycolytic and OPP pathways (Kumaraswamy et al., 2013). In the former, one molecule of G6P can provide two molecules of NADH whereas seven molecules of reduced pyridine nucleotides can be produced from the same molecule of G6P (6 NADPH per glucose and one NADH per glucose) *via* the OPP pathway (Kumaraswamy et al., 2013). One molecule of NAD(P)H can be oxidized by H_2_ase to provide one molecule of H_2_. Therefore, the reduced pyridine nucleotides from the OPP pathway can produce more H_2_ compared with that from the glycolytic pathway (seven molecules vs. two molecules of H_2_). In plentiful conditions or in the case of the BG11 (Figure 5B (+NK)), cells were more likely to generate organic acid compounds than potassium-deprived adapted cells were. This corresponded with a previous study of *Synechocystis* sp. PCC 6803 that suggested that, under dark anaerobic conditions, where NADPH was limited, there was a competing demand to consume NADPH for H_2_ and organic acid production. In the presence of potassium, cells preferred to utilize NADPH for production of organic acids (Ueda et al., 2016). Therefore, NAD(P)H is normally used for organic acid production and the nitrate assimilation pathway rather than H_2_ production. In nitrogen-deprived cells, it may be that glycogen catabolism occurs *via* both the OPP and glycolytic pathway, as shown in *Synechocystis* sp. PCC 6803 (Osanai et al., 2006). In potassium-free cells, PK activity (Figure 3) and the gene expression of *pyk* encoding PK was lower than that found in normal cells (Figure 4). Consequently, glycogen was mainly degraded *via* the OPP pathway and provided more NADPH for H_2_ production.

## 5 Conclusion

Under dark anaerobic conditions, the maximum H_2_ production of 1,261.96 ± 96.99 µmol H_2_ g dry wt^−1^ and the maximum hydrogenase activity of 179.39 ± 8.18 µmol H_2_ g dry wt^−1^ min^−1^ was found in *A. halophytica* cells incubated in the nitrogen- and potassium-deprived BG11 medium supplemented with Turk Island salt solution for 48 h. The increased hydrogenase activity was due to a reduction of O_2_ in the system, resulting from the lower photosynthetic O_2_ evolution and higher dark respiration. Under nitrogen and potassium deprivation, *A. halophytica* cells promoted the production and accumulation of glycogen and reduced pyruvate kinase activity. Transcriptional analysis by RNA-seq helped to understand the effect of nitrogen and potassium deprivation on H_2_ production by *A. halophytica*. Several genes involved in glycogen biosynthesis (*glgA*, *glgB*, and *glgP*) were upregulated in both nitrogen- and potassium-deprived cells. However, *pyk* and most genes that regulated enzymes in the glycolytic pathway were down-regulated in both nitrogen- and potassium-deprived cells. Interestingly, most genes that regulated enzymes in the oxidative pentose phosphate pathway (OPP) were upregulated. Accordingly, the OPP was suggested as a promising pathway for enhancing H_2_ production under dark anaerobic conditions in both nitrogen- and potassium-deprived *A. halophytica* cells. This study indicated that combined nitrogen and potassium deprivation in media is a promising strategy to promote sustainable H_2_ production by the halotolerant cyanobacterium *A. halophytica.*

## Data Availability

The original contributions presented in the study are included in the article/SupplementaryMaterial; further inquiries can be directed to the corresponding author.
